# Clinical audit of veterinary professionals’ cardiopulmonary resuscitation (CPR) skill and knowledge retention within a small animal hospital

**DOI:** 10.18849/ve.v9i4.674

**Published:** 2024-12-05

**Authors:** Clare Jones

**Keywords:** arrest, cardiopulmonary resuscitation, education, knowledge, retention, skill, small animal, training

## Abstract

**Introduction:** The RECOVER guidelines (2012) on veterinary cardiopulmonary resuscitation (CPR) suggest refresher training every 3–6 months. This audit was undertaken to see if this can impact veterinary professionals’ psychomotor skills and knowledge retention. Since June 2022, within Hale Veterinary Group small animal hospital, a VetPartners practice in the UK, staff members’ knowledge was audited using a multiple-choice questionnaire (MCQ) and their skills were assessed using a CPR training cube to monitor compression rate and recoil.

**Aims and objectives:** To see over what time period psychomotor skills and knowledge of CPR are most commonly lost.

**Background:** Aiming to improve the knowledge and psychomotor skill retention of veterinary professionals, as well as frequency of training. Following RECOVER guidelines (2012) suggestion of refresher training, looking at NHS training suggestions and audits, to improve knowledge and skill retention.

**Methods:** To measure knowledge retention a MCQ was created, based on the RECOVER basic life support and advanced life support components. Looking at similar MCQs within human medicine, the decision was made to keep the MCQ anonymous but to categorise participants into job roles. To measure psychomotor skills a device (cprCUBE PRO) was bought by the practice to monitor compression rate and recoil abilities.

**Results:** Results showed that veterinary surgeon and registered veterinary nurse knowledge and skill retention is best maintained with refresher training every 6 months to 1 year.

**Implementation of changes (Team discussion & changes made):** Training to be offered every 3 months, clinical staff to attend training every 6 months to 1 year.

**Re-audit:** The initial training session was held over one day. Following the results of the audit, the subsequent September 2022 training session was held over two days in an attempt to improve attendance. This session was then audited.

**Application:** Clinical staff must attend a training session within the year, new members of staff to attend the soonest possible training session. Other practices would benefit from having training and implementing frequent refresher training.

## Introduction

As clinical governance and quality improvement encourages veterinary staff to look at improving practice and care provided, I have looked at the implementation of cardiopulmonary resuscitation (CPR) training within a practice. The RECOVER guidelines (Fletcher et al., 2012) advise retraining of CPR at least every 6 months as well as cardiopulmonary arrest scenario ‘mock codes’ every 3–6 months. The aim of this clinical audit is to see how frequent training might impact knowledge and psychomotor skill retention within staff. This is important as evidence has shown that without regular training of CPR, both skills and psychomotor skills can diminish over time (Nori et al., 2012; and Sand et al., 2021). Although there are no studies to show that this can benefit veterinary professionals, as it is staff performance being audited, the human medicine studies can be relevant. The veterinary hospital this audit started at had previously logged 21 cardiopulmonary arrests from January 2019 to June 2022; four patients were successfully resuscitated, with only one patient surviving to discharge. In a survey by Gillespie et al. (2016) it was found that 1.6–6% of dogs and 2.3–9.6% of cats survived a cardiopulmonary arrest event within a veterinary hospital. This is notably lower than the adult human survival to discharge rate of 24% (Mozaffarian et al., 2016). In human medicine the ability to perform adequate CPR can contribute to patient survival rates (Oermann et al., 2012). Although there are few studies into frequency of veterinary CPR training and patient survival rates, increased frequency of CPR training may improve patient outcomes.

This study was a process clinical audit, looking at whether the CPR training protocol is achieved and managed appropriately.

## Methods

Simulation training was used to create a ‘crash scenario’, to measure psychomotor skills used in CPR, and cprCUBE PRO (https://cprcube.com) was used to monitor participants’ chest compression rate and recoil reflexes. Multiple-choice questionnaires (MCQs) are often used in human medicine to monitor knowledge retention. To measure knowledge for this audit, an MCQ was created by two registered veterinary nurses (RVNs) within the practice after researching CPR training MCQs in human medicine (see Supplementary material 1). This MCQ was completed prior to each training session by the participants, who included veterinary surgeons, RVNs, student veterinary nurses (SVNs), and animal care assistants (ACAs). There were 75 attendees in total over the four training sessions. At the first session (5 July 2022) there were 24 participants, with 15 at the second and third sessions (30 September 2022, 30 December 2022), and 21 at the fourth session (9 March 2023). These results were recorded and audited to monitor for improvement of both psychomotor skills and knowledge retention. The cprCUBE PRO is an electronic machine that simulates chest compressions. It records compression rate, recoil ability, and depth of the compressions performed by those using it. The cprCUBE PRO is linked directly to a smart tablet for the assessor to be able to monitor the participants’ abilities. The results were then shown to the participant after they had performed the chest compressions. The staff member (Clare Jones) who led and assessed the training in-house received RECOVER CPR basic life support and advanced life support training to become a RECOVER rescuer able to provide the most up-to-date information to the team.

Training was arranged every 3 months within the practice, and the MCQ was taken prior to any training session to measure knowledge retention. The psychomotor skills of staff performing chest compressions were also measured prior to training, recording compression rate and recoil ability using the cprCUBE PRO, to measure skill retention.

The training provided followed the RECOVER guidelines (2012) using the RECOVER algorithm, training staff to perform chest compressions at a rate of 100–120 compressions per minute.

## Results

Staff were asked if they felt their knowledge had improved since training. 87% (13/15) of all participants in the second training session answered that they felt their knowledge and psychomotor skills had improved and retained since their last CPR training session. This has helped with staff feeling better prepared for arrests and more positive regardless of outcomes. During post-CPR debriefs staff have reported that they were all competent following training.

**Figure 1:**
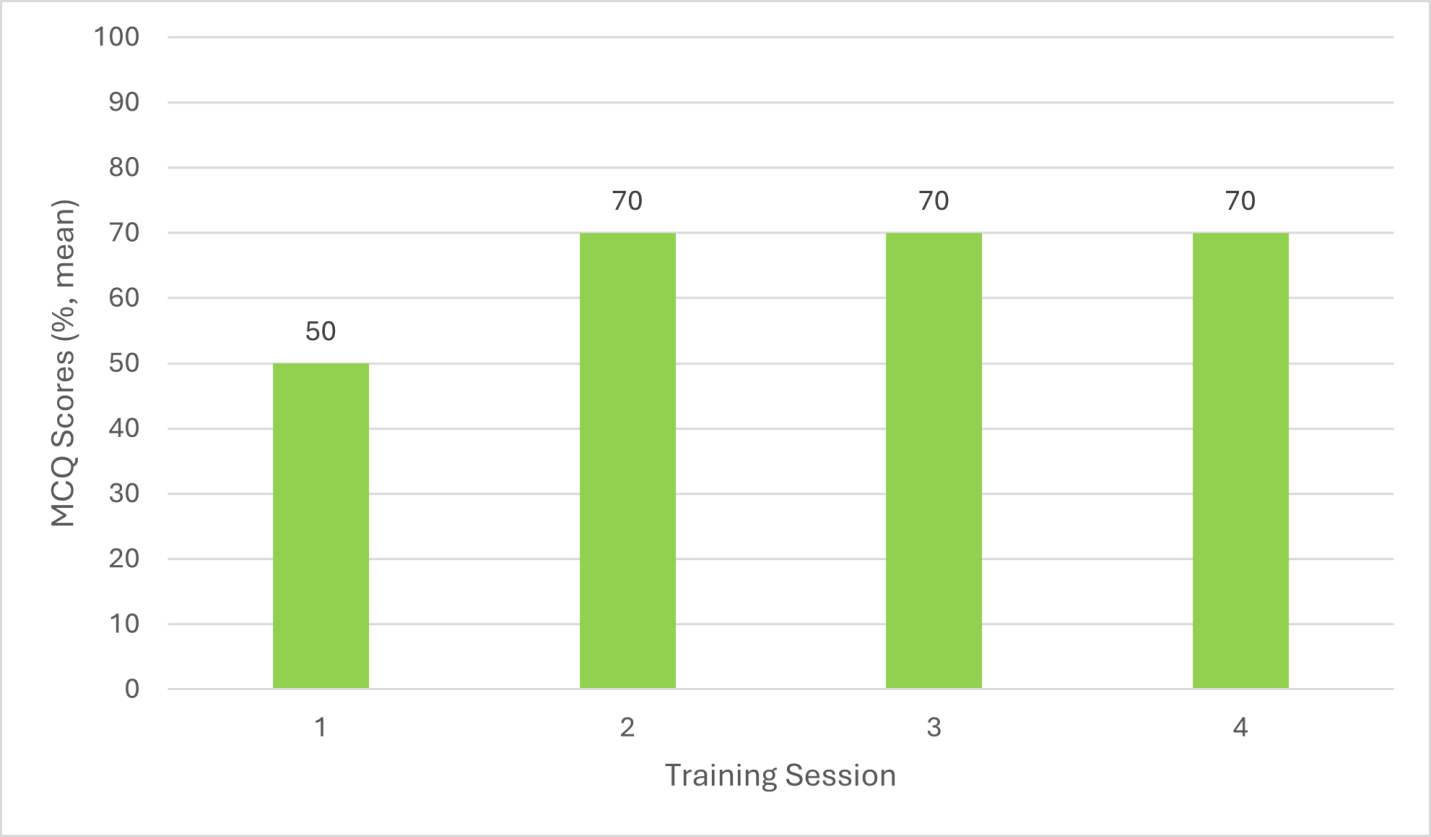
Knowledge retention of veterinary surgeons over four training sessions. Figure shows average MCQ scores from participants in each training session.

**Figure 2:**
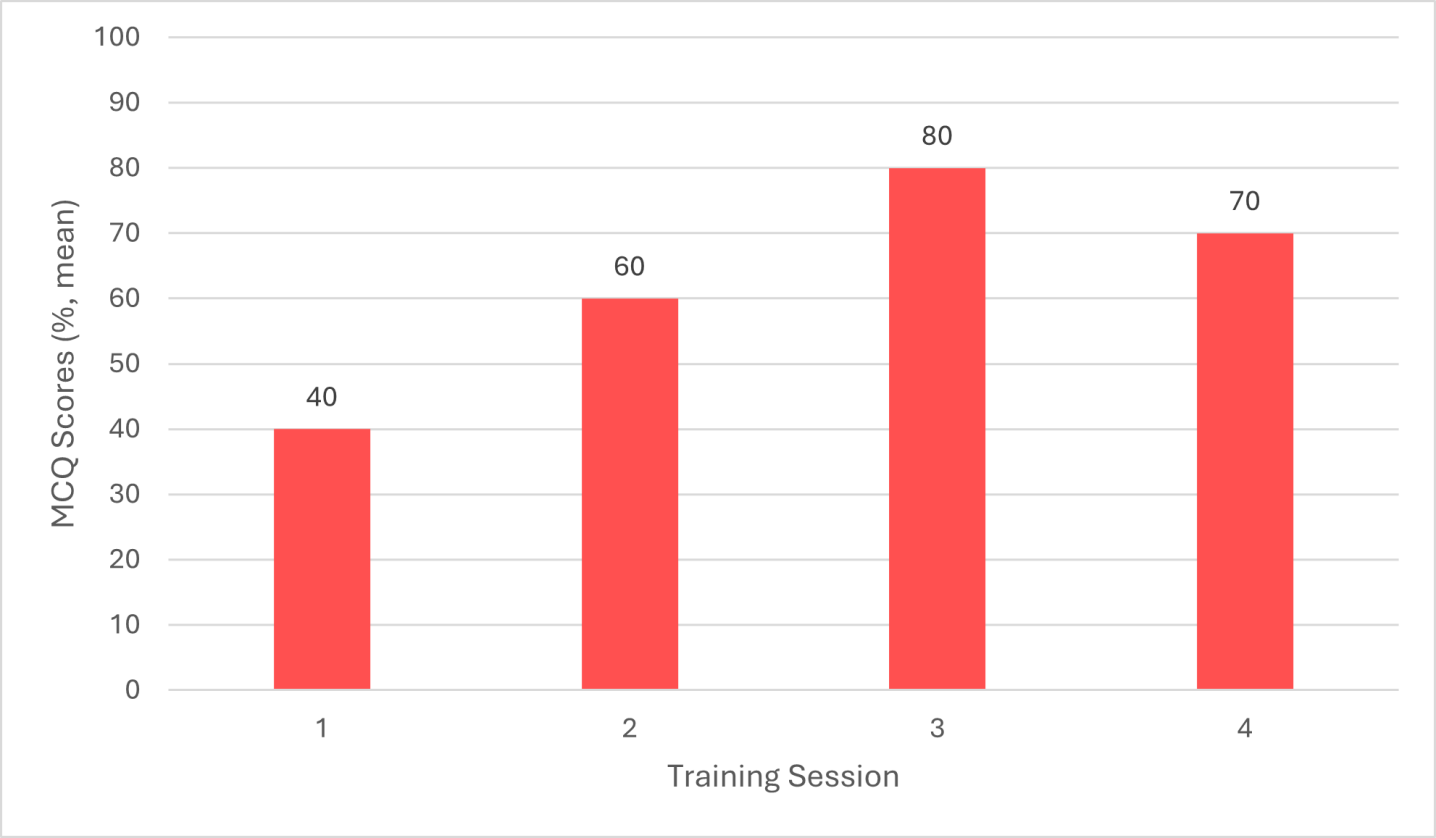
Knowledge retention of veterinary nurses over four training sessions. Figure shows average MCQ scores from participants in each training session.

**Figure 3:**
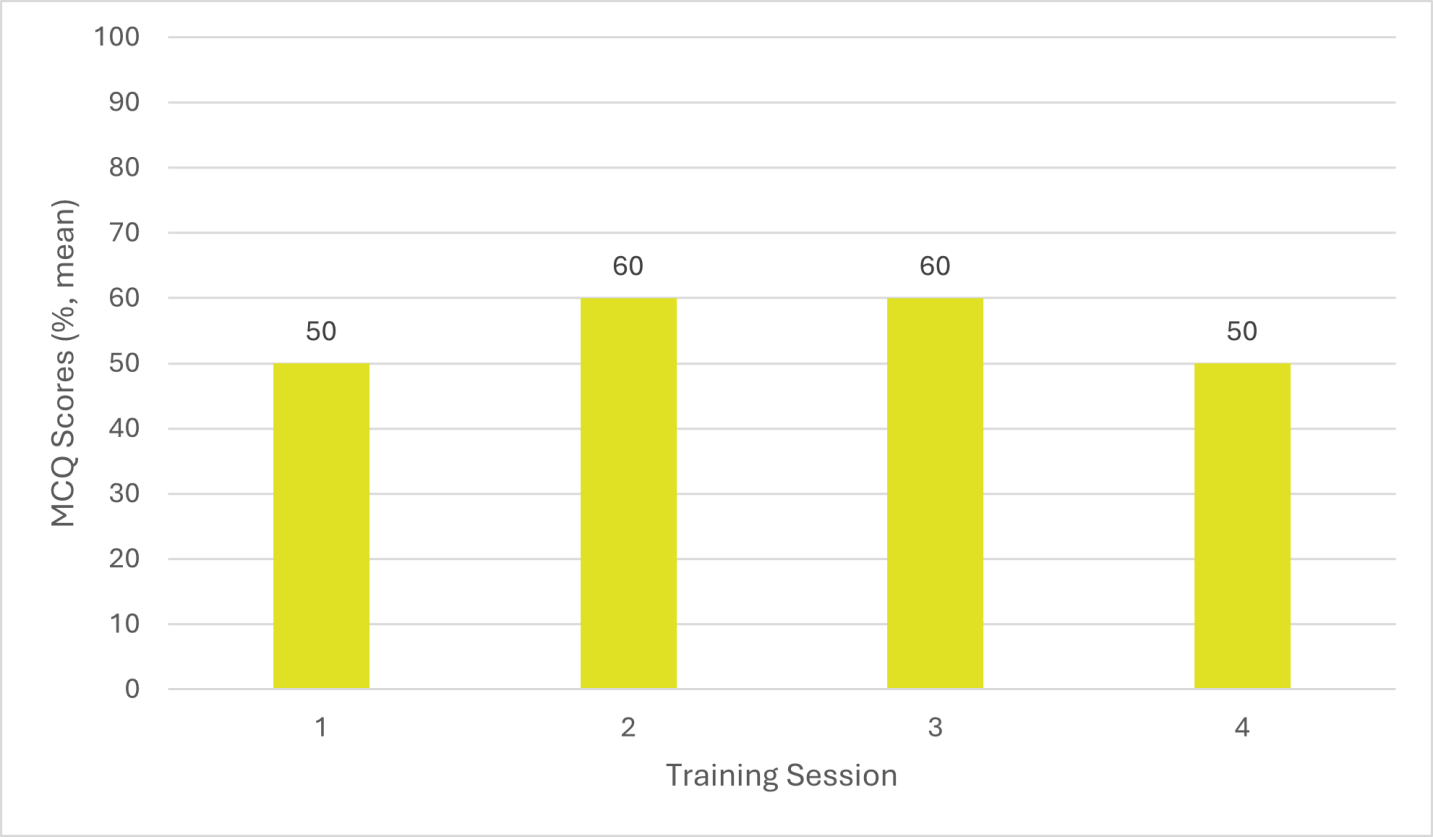
Knowledge retention of student veterinary nurses and animal care assistants over four training sessions. Figure shows average MCQ scores from participants in each training session...

MCQ results showing participants’ CPR knowledge prior to the first training session showed that veterinary surgeons answered on average 50% (n = 9) correctly, RVNs answered on aver- age 40% (n = 10) correctly, and SVN/ACAs answered on average 50% (n = 5) correctly (Figures 1 to 3). Before the second session, held 3 months after the initial training, veterinary surgeons answered on average 70% (n = 6) correctly, RVNs 60% (n = 6) correctly and SVN/ACAs 60% (n = 3) correctly. Prior to the third session, held 6 months after the initial training, veterinary surgeons answered on average 70% (n = 3) correctly, RVNs 80% (n = 8) correctly, and SVN/ ACAs 60% (n = 2) correctly. Finally, prior to the fourth session, held 9 months after the initial training session, veterinary surgeons answered on average 70% (n = 5) correctly, RVNs 70% (n = 11) correctly, and SVN/ACAs 50% (n = 4) correctly. This shows a marked improvement in knowledge with regular training.

**Figure 4:**
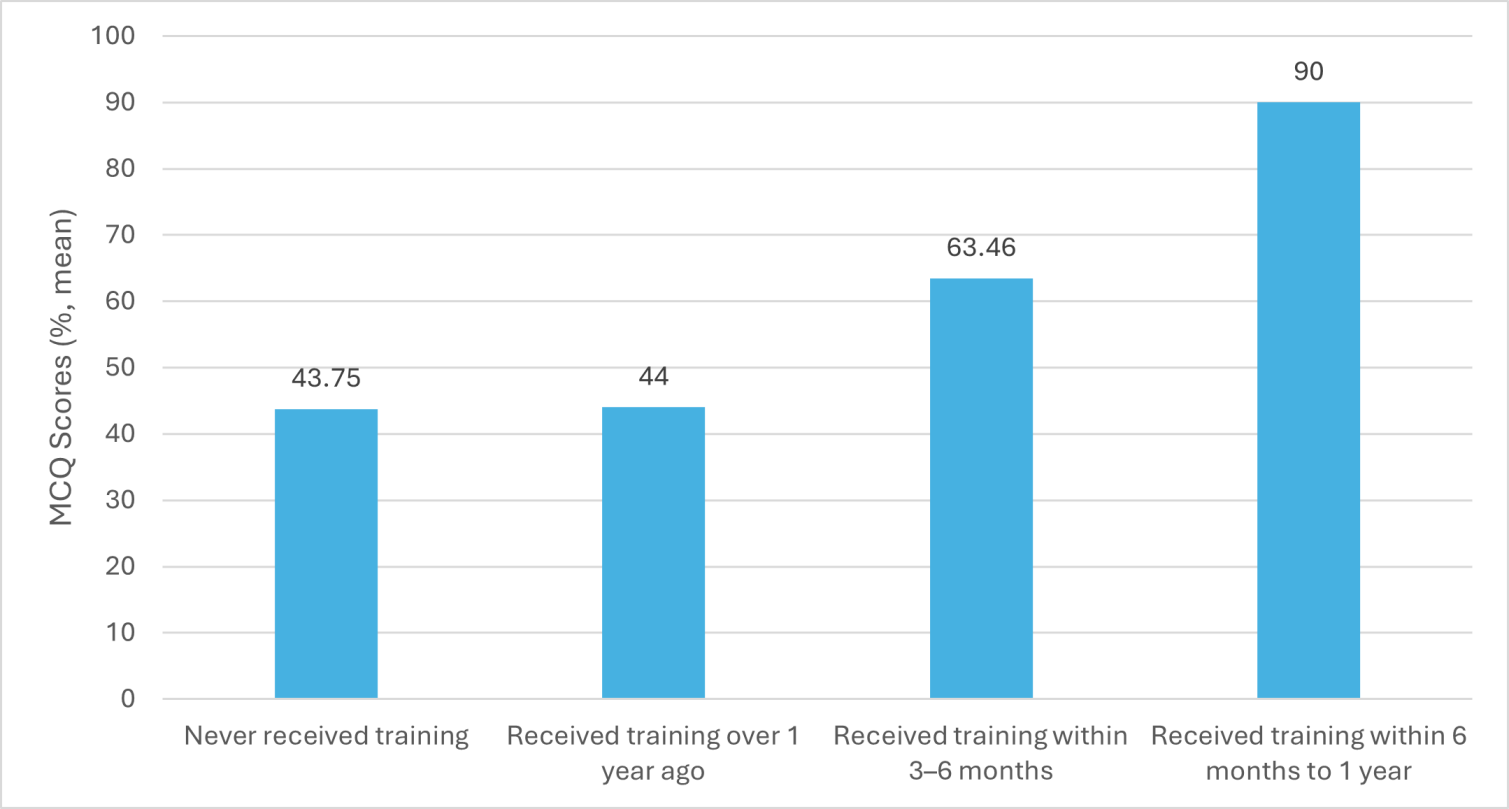
Average MCQ scores for participants grouped according to when they last received CPR training.

Of the 24 participants, the 14 who had never had training prior to the first audit averaged 43.75%, and the 9 who had training over a year ago averaged 44% (Figure 4). This increased at the second audit, with those who had received training within 3–6 months answering on average 63.64% correctly, and again in the third audit, with those who received training within 3–6 months answering on average 66.92% correctly and those who received training within 6 months to 1 year answering on average 90% correctly. This would demonstrate a need for CPR training to retain knowledge to be taken every 3–6 months for the best results.

**Figure 5:**
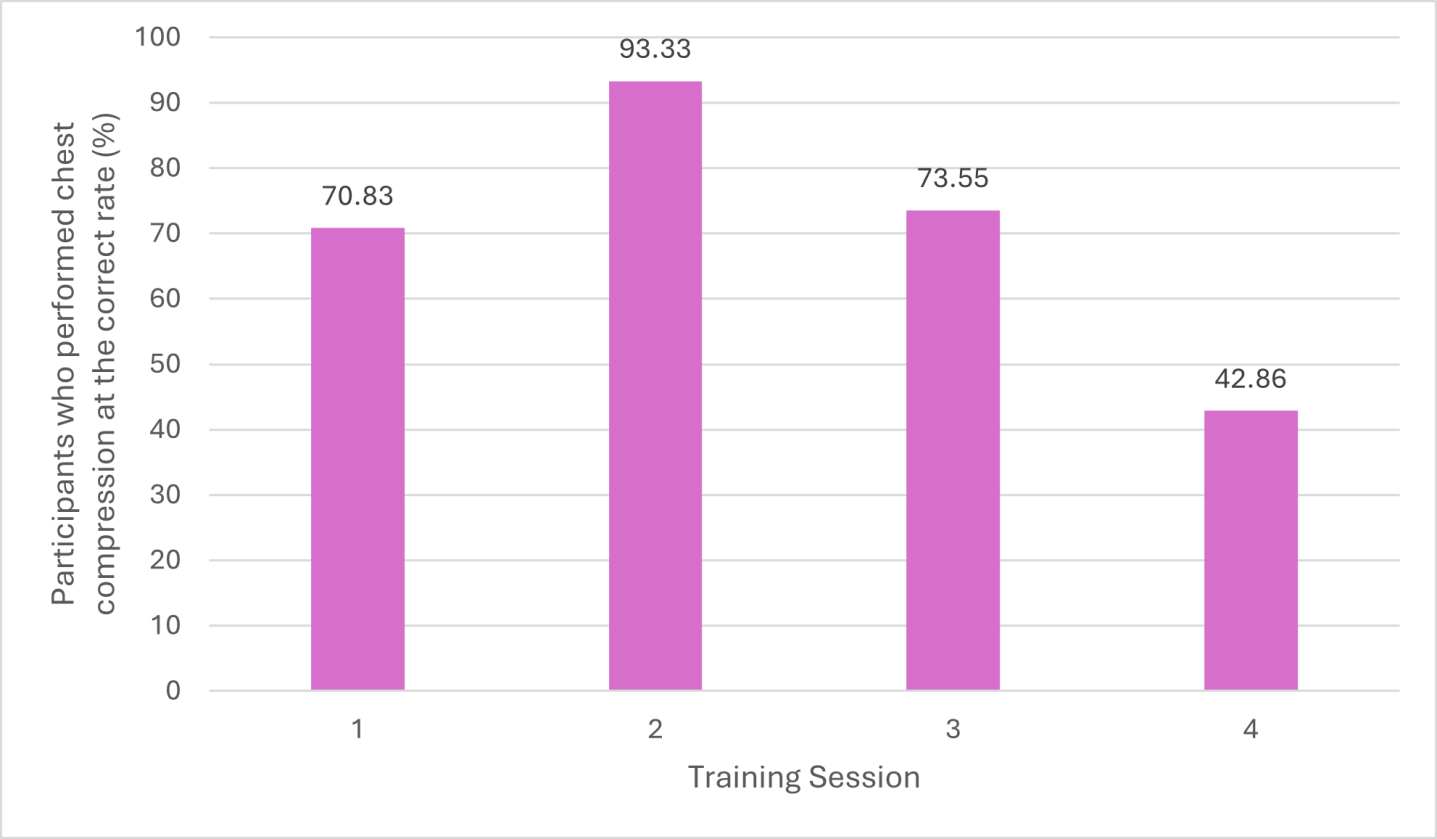
Participants’ ability to perform chest compressions at the correct rate (100–120 cpm). Figure shows average scores from participants in each training session.

During the first training session 70.83% (17/24) of participants performed chest compressions on the cprCUBE PRO at the correct rate, and this improved at the second training session to 93.33% (14/15) (Figure 5). The third session showed a result of 73.33% (11/15) per- formed at the correct rate, followed by a large decrease in accuracy in the fourth session to 42.86% (9/21). This variation over the four training sessions may indicate a need for more frequent training to ensure that psychomotor skills can be retained for CPR performance.

**Figure 6:**
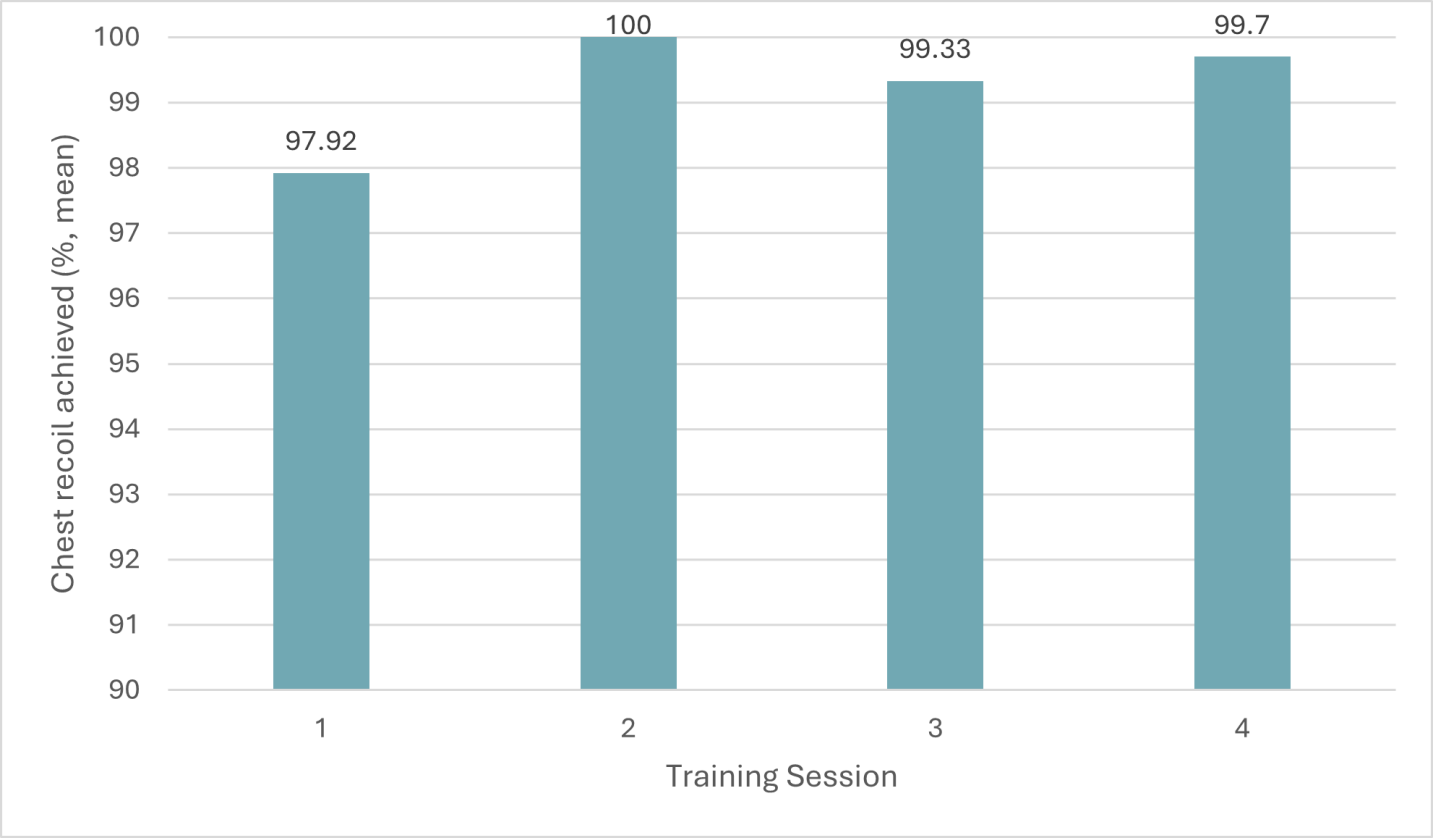
Participants’ ability to allow full chest recoil during chest compression training. Figure shows average scores from participants in each training session.

Ability to achieve 100% chest recoil was recorded over the four training sessions (Figure 6). Participants averaged 97.92% in the first session, 100% in the second session, 99.33% in the third session, 99.7% in the fourth session. These results remained similar over the course of the four training sessions. The desired chest recoil is 100%, so to achieve this consistently more frequent training may be required to ensure these psychomotor skills are also retained.

**Recommendations:** This audit would benefit from more practices being involved to get more varied data collection. However, the results for this practice knowledge retention and psychomotor skills show a need for staff to have training every 3–6 months.

**Action plan:** The audit within this particular practice will continue to be carried out every 3–6 months. The hope is to advertise this audit to other practices to encourage them to take part.

## Implementation of changes (team discussion & changes made)

Clinical staff have been encouraged to attend training every 3–6 months, following the RECOVER guidelines (2012). To improve veterinary surgeon attendance, it was decided to provide refresher training over 2 days every 3 months. This change would enable staff to have two days to choose from to attend, meaning they were less restricted with dates and times, which would ease attendance. It was also arranged to offer 1-day training at the practice’s three branch surgeries, to improve attendance of staff based at a branch.

## Re-audit results

Offering the training over 2 days every 3 months, instead of 1 day every 3 months, helped with staff attendance and availability, allowing staff to have the option and flexibility of two days and times to attend training. As well as this running the CPR training at each branch also improved the attendance greatly. Staff attendance had dropped following the initial training, then increased greatly on the fourth session (24 attendees in the first session, 15 in the second and third sessions, 21 in the fourth session). This was with training being offered at the three-branch practice, which helped not only increase attendance but also give a more varied result of staff taking part, different veterinary surgeons and RVNs with varying experience and previous training.

## Conclusion

The audit into CPR training made staff feel more confident after the training, which then led to improving the hospital and branch practices’ crash box (veterinary emergency kit) and cardiopulmonary arrest protocols. Staff attendance became less after the initial training session, decreasing from 24 to 15 attendees. This was due to time restrictions and staff compliance. RVN and SVN/ACA attendance was more easily retained compared with veterinary surgeons. This was reflected within the MCQ scores, with RVNs achieving on average 80% (n = 8) and 70% (n = 11) in the third and fourth sessions, and veterinary surgeons answering on average 70%% (n = 3) and 70% (n = 5) in the third and fourth session. In the initial session, RVNs scored an average of 50% (n = 9) and veterinary surgeons scored 40% (n = 10). Two training sessions every 3 months on different days were organised to try improving attendance from all staff. From the results it is clear to see that training should be undertaken by staff every 6 months to 1 year, but having training available every 3 months may provide more opportunities for staff to attend and to train any new members of staff.

Those participants who had never had training prior to the first session achieved on average 43.75% (4.37/10) on the MCQ, and those who had training over a year ago averaged 44% (4.4/10). This increased by the second session for those who had had training within 3–6 months averaged at 63.64% (6.36/10) MCQ questions answered correctly, again in the third session those with training within 3–6 months averaged 66.92% (6.69/10) answered correctly and those within 6 months to 1 year achieved on average 90% (9/10) correctly answered. This would demonstrate a need for CPR training to retain knowledge to be taken every 3–6 months for the best result. The psychomotor skills auditing initially showed 66.67% (16/24) of participants were performing the correct chest compression rate (100–120), this improved to 86.67% (13/15) achieving the correct rate of chest compressions by the second session. Participants’ ability to allow for full recoil of chest during chest compressions showed staff averaged 97.92% (23.5/24) this reached 100% (15/15) by the second session. These results show an improvement in psychomotor skills within the staff after initial training, but this did reduce over the last two training sessions, this reinforces the need for regular training to ensure skills are maintained.

Patient survival outcomes are difficult to audit in cardiopulmonary arrest within veterinary medicine, with lots of different contributing factors to consider. Since January 2019 to March 2023 the practice has recorded 23 attempts at CPR. Prior to frequent training starting on June 2022, 15 attempts at cardiac resuscitation have been recorded with no patients surviving to discharge, since implementing frequent training in June 2022 eight attempts at CPR have been recorded with one patient successfully surviving to discharge. However, this audit has only been done within one practice and would be more reliable with a larger number of patients, as well as looking at in-hospital and out-of-hospital arrests, including those under anaesthetic or not. An extended more focused audit would be beneficial here.

Since starting the initiative and training within the practice, the team have been enthusiastic, and keen to learn and improve their clinical skills. The practice supplied training equipment to conduct the audit and training, and Clare Jones and a nursing manager created the MCQs to ensure relevancy. Alongside the training the practice also supplied updated CPR protocols, including emergency drug doses for a variety of species and the RECOVER CPR algorithm.

The validity of the audit is poor as there is little research into CPR training within small animal veterinary professionals, and this is hard to improve. The use of MCQs within human medicine helps the reliability of the use of MCQ in this audit, adapting the questions to fit within the small animal practice they were used for. However, the use of the cprCUBE PRO is unreliable as it is a training tool designed for human resuscitation not veterinary companion animals. For this reason, the aspects measured from the cprCUBE PRO were chest compression rate and recoil, as these were easily comparable to small animal patients. This audit will continue within Hale Veterinary Group Hospital and has been incredibly interesting and rewarding. The hope is that more practices will take part in this audit to gather more data.

## Application

Results have been shared with staff and frequent training for clinical staff is now mandatory and it has been offered out to other nearby VetPartners practices also. RECOVER guidelines (2012) suggest training every 3–6 months, but these results show that knowledge and skills are retained well between 6 months and 1 year after training. These results should be used by veterinary professionals working within clinical settings, and they should share these results to their practice to demonstrate to them the need for frequent training within practice. This audit will be ongoing within the practice to continuously improve standard of training and care for patients. In addition to this audit, Clare Jones audited patients post cardiopulmonary arrest survival rate. This audited the patients who received CPR following an arrest to see if they survived to discharge, in comparison to successful resuscitation and later either being euthanised or dying in hospital. The purpose of this was to evaluate whether frequent training could improve patient outcome. Over the period of January 2019 to October 2022, Hale Veterinary Hospital’s current post cardiopulmonary arrest survival rate is 17.39% (4/23), however, the survival to discharge rate is 4.34% (1/23).

## Informed consent

All staff that participated understood that their data would be anonymised and audited to understand where improvement may be needed. Staff who repeated the training sessions were asked at each training session to consent to use the data.

## Supplementary materials

Supplementary material S1 – Supplementary 1: Multiple-choice questionnaire used during the audit for training, 11 questions to be answered correctly.

## Acknowledgements

The author would like to thank Nicole Archer-Fairley for assistance with designing the questionnaire.

## ORCID

Clare Jones: https://orcid.org/0000-0003-1550-6285

## Conflict of Interest

The author declares no conflict of interest.

## Intellectual Property Rights

Authors of articles submitted to RCVS Knowledge for publication will retain copyright in their work, and will be required to grant to RCVS Knowledge a non-exclusive licence of the rights of copyright in the materials including but not limited to the right to publish, re-publish, transmit, sell, distribute and otherwise use the materials in all languages and all media throughout the world, and to licence or permit others to do so.

## Disclaimer

Any opinions expressed in articles and other publication types published in Veterinary Evidence are the author's own and do not necessarily reflect the view of the RCVS Knowledge. Veterinary Evidence is a resource to help inform, and the content herein should not override the responsibility of the practitioner. Practitioners should also consider factors such as individual clinical expertise and judgement along with patient’s circumstances and owners’ values. Authors are responsible for the accuracy of the content. While the Editor and Publisher believe that all content herein are in accord with current recommendations and practice at the time of publication, they accept no legal responsibility for any errors or omissions, and make no warranty, express or implied, with respect to material contained within. For further information please refer to our Terms of Use.
